# Psychopathology in Dutch women with terrorist behaviours: empirical case series study

**DOI:** 10.1192/bjo.2025.9

**Published:** 2025-04-16

**Authors:** Sadaf Rakhshandehroo, Nils Duits, Lieke van Emmeriek, Elise Pullen, Robbert-Jan Verkes, Maaike Kempes

**Affiliations:** Department of Science and Education, Netherlands Institute of Forensic Psychiatry and Psychology, Utrecht, The Netherlands; Donders Institute for Brain, Cognition and Behaviour, Radboud University, Nijmegen, The Netherlands; Department of Criminal Law and Criminology, Radboud University, Nijmegen, The Netherlands; Faculty of Social and Behavioural Sciences, Leiden University, Leiden, The Netherlands

**Keywords:** Female terrorism, psychopathology, mental disorder, forensic mental health assessment, empirical research

## Abstract

**Background:**

Current empirical understanding of the relationship between psychopathology and terrorist behaviours in women is limited, because most research focuses on male perpetrators and relies on secondary sources. Addressing this gap is crucial, particularly given previous research that highlights significant differences in mental health problems between women and men involved in non-terrorist violent activities.

**Aims:**

To empirically examine the presence of psychopathology in women exhibiting terrorist behaviours, as well as its potential role in these behaviours.

**Method:**

A case series study of 14 Dutch female convicts associated with the (so-called) Islamic State of Iraq and Syria (ISIS), examining the occurrence and types of mental disorders, psychopathological problems and pathological personality traits, and exploring their potential role in terrorist behaviours based on forensic mental health reports from psychiatrists and psychologists.

**Results:**

Half of the women (*n* = 7) exhibited mental disorders during terrorist activities, primarily personality disorders. Psychopathological problems included susceptibility to influence (71%, *n* = 10), identity problems (64%, *n* = 9), feelings of inferiority (57%, *n* = 8) and naivety (50%, *n* = 7). A significant link between terrorism and mental disorders, psychopathological problems or pathological personality traits was identified in almost half of the women (43%, *n* = 6).

**Conclusions:**

Psychopathology is present in some women involved in terrorist behaviours, influencing their involvement, but is absent or irrelevant in others. Identifying psychopathology in women with terrorist tendencies is essential for early prevention and should be a core competency for psychiatrists.

Terrorist acts have far-reaching global consequences, including loss of life, emotional trauma, material destruction, economic repercussions and potential threats to democratic values.^1–4^ Investigating the underlying risk factors of terrorism, with the ultimate goal of obtaining targets for prevention, is therefore a vital scientific pursuit.^5^ One of the risk factors associated with terrorist behaviour is mental health problems in perpetrators. However, studies on psychopathology in individuals with terrorist behaviours have predominantly focused on male perpetrators, often marginalising female perpetrators as ‘statistically insignificant outliers’^6^ (p. 41). The scarcity of female-specific knowledge on this topic, combined with evidence of gender-specific distinctions in psychopathology among (non-terrorist) violent individuals,^7–10^ underscores the need for further exploration in women with terrorist behaviours. This understanding is pivotal for tailoring counter-terrorism strategies and mitigating the risk of recidivism.^11^ Examining the connection between psychopathology and terrorist behaviours in female perpetrators, our study reveals unique and empirically based insights into this complex relationship by investigating the presence and role of psychopathology in a case series study of Dutch female terrorist offenders associated with the (so-called) Islamic State of Iraq and Syria (ISIS).

## Current empirical understanding of psychopathology in terrorism

Before exploring empirical knowledge on psychopathology in perpetrators of terrorism, it is important to note the following key points. First, there is no empirical evidence suggesting that terrorism is predominantly committed by individuals with mental illness.^12,13^ Moreover, when mental illness is present, its relevance to engaging in terrorist behaviours may be limited or even protective.^14^ In cases where a mental disorder is relevant, it often plays a functional or contextual role rather than a causal one, and it is influenced by political, ideological, situational and biopsychosocial factors. Given these nuances, understanding the role of psychopathology in terrorism remains important.

Delving deeper into the current empirical understanding of psychopathology in terrorist populations, several systematic reviews have explored this topic. According to Sarma and colleagues,^12^ the lifetime prevalence of diagnosed mental disorders in predominantly male terrorist samples is 17%, which increases to 26% when studies including both suspected and diagnosed mental disorders are considered. Regarding the connection between psychopathology and terrorist behaviours, however, this review concludes that the majority of studies thus far are hindered by methodological challenges. Studies often rely on open sources rather than robust examinations of the perpetrators, are quantitative rather than qualitative and exhibit a high risk of bias.^12^

Similarly addressing these complexities, Gill et al^13^ identified prevalence rates of detected mental health issues in violent extremist populations ranging from 0 to 57%. When considering studies with confirmed diagnoses, the prevalence rate was 14%, slightly increasing to 17% in studies accessing more privileged sources such as police or judicial data. In line with the systematic review by Sarma et al,^12^ the significance of psychopathology in terrorist behaviours has remained underexplored according to this review, due to the same methodological limitations prevalent in the majority of the published literature.

## Empirical research on psychopathology in female perpetrators of terrorism

Another notable gap in research on psychopathology in terrorist populations is the underrepresentation of female perpetrators, resulting in scarce primary studies within this group. Among studies that do include women in predominantly male samples, few differentiate findings by gender, thus hindering insights into female-specific psychopathological issues.^15^ While limited, available female-specific data suggest indications of personality disorders and suicidality among women involved in terrorism.^15^ The importance of exploring psychopathology in female terrorist perpetrators is further underscored by empirical insights into gender disparities in mental health problems among violent non-terrorist offenders. Women involved in violence often exhibit higher rates of internalisation and dissociative disorders, with lower rates of externalisation disorders compared with men. They also show higher levels of comorbidity and engagement in self-harming behaviours.^7–10,16,17^

The importance of studying psychopathology in female perpetrators of terrorism has increased since the participation of women in ISIS.^18^ Since mid-2014, ISIS has involved women in roles such as ideological enforcement, propaganda, recruitment and support. With some women repatriated, policymakers are concerned about potential recidivism. Understanding whether mental health problems have played a role in their involvement, and how these issues have influenced their terrorist behaviours, are crucial for developing empirically based, tailored counterterrorism strategies and mitigating future risks among these and other at-risk women.

Empirical research on psychopathology in perpetrators as a risk factor for terrorist behaviour has been facilitated by the European Database of Terrorist Offenders (EDT), established in 2012.^19^ The EDT is an innovative research database that aggregates extensive judicial information on European convicted terrorist offenders, encompassing their demographics, childhood circumstances, triggers, ideologies, motives and mental health problems potentially related to engagement in violent extremism and terrorism. One of the primary sources of data integrated in the EDT is forensic mental health reports, derived from comprehensive assessments by psychologists and psychiatrists, which offer crucial insights into the potential link between psychopathology and terrorism. The EDT is realised by a collaboration among European Union judicial bodies and scientists from The Netherlands, Belgium, Germany, Austria and Sweden, and is managed by the Netherlands Institute of Forensic Psychiatry and Psychology (NIFP).

## Study aim

Our study aimed to explore the relationship between psychopathology – i.e. mental disorders, psychopathological symptoms or pathological personality traits – and terrorist behaviours in Dutch female offenders associated with terrorism. Drawing on forensic mental health assessments by psychiatrists or psychologists, we sought to investigate whether psychopathology influenced these behaviours and, if so, how and to what extent. The following research questions were addressed: (a) were mental disorders present at the time of engaging in terrorism among Dutch female convicts and, if so, which types? (b) Were psychopathological symptoms or pathological personality traits present during this period and, if so, which types? (c) To what extent did psychopathology contribute to their offences? (d) How did psychopathology play a role in their terrorist behaviours? We included psychopathological symptoms and pathological personality traits alongside mental disorders to avoid oversimplifying the terrorist population into dichotomous categories of mentally ill or non-ill.^20–22^ Definitions followed the Diagnostic and Statistical Manual of Mental Disorders, 4th^23^ or 5th^24^ edition, depending on the version used in our data.

## Method

### Study sample

Dutch female convicts of terrorism who underwent pre-trial forensic mental health assessments were to be included in the study sample. To assemble the group, a list of Dutch convicted female terrorist offenders was requested and obtained from the NIFP’s EDT project team (see ‘Ethics approval’). This resulted in the inclusion of all 14 Dutch women in the EDT (mean age 22.21 years, s.d. 5.69). These women were convicted of ISIS-related terrorism offences between 2012 and 2022. For our study, we included only those terrorist offences for which the women were convicted, excluding alleged terrorist offences that were not proven and for which the women were acquitted by the court. This approach ensured that our focus remained on the role of psychopathology in the confirmed terrorist behaviours of these women. To determine the terrorist offences for which the women were convicted, we reviewed their verdicts on the Dutch government’s publicly available judiciary website (https://www.rechtspraak.nl/), using court case numbers provided by the Department of Science and Education of the NIFP. This website provides information about legal procedures, verdicts and the organisation of the judiciary in The Netherlands. In three female subjects, it became apparent that not all the alleged offences were proven, and these unproven terrorist acts were excluded from this study. Table 1 details the terrorist offences and specific terrorist behaviours for each case involving a convicted female, with ‘participation in a terrorist organisation’ being the most common offence (*n* = 10).

**Table 1:** Terrorist offences and behaviours of Dutch female terrorist offenders (*N* = 14)

Subject number	Terrorist offences						
	Participation in a terrorist organisation	Preparation for murder, manslaughter, intentional arson, explosion, occasion, means or intelligence, with terrorist intent	Attempted participation in a terrorist organisation	Possessing prohibited weapons	Providing opportunity, resources, intelligence, knowledge or skills for terrorist crime	Incitement to commit terrorist offences	Complicity in murder or manslaughter and initiating an explosion with terrorist intent
1		+^a^					
2	+^b^						
3	+^c^						
4	+^d^			+^d^			
5	+^e^						
6			+^f^				
7			+^g^				
8	+^h^	+^h^					
9	+^i^			+^i^			+^i^
10	+^j^	+^j^					
11	+^k^	+^k^					
12			+^l^				
13	+^m^				+^m^	+^m^	
14	+^n^	+^n^					

Data are based on information obtained from the official Dutch judiciary website regarding trial procedures and verdicts in The Netherlands (https://www.rechtspraak.nl/).

a. During her citizenship in the Islamic State of Iraq and Syria (ISIS) region, she was aware of her husband’s participation in ISIS onsite, and was aware of the gruesome terrorist offences committed by ISIS in Syria and Iraq, thereby facilitating her husband.

b. She travelled to Syria, joined ISIS, married an ISIS fighter and formed a family with him, received monthly wages from ISIS, regularly stored weapons provided by ISIS for her husband at home and, after her husband’s death, received a one-time widow’s allowance for ISIS fighters.

c. She travelled to the area of the ISIS caliphate, pledged allegiance, accepted financial compensation from ISIS, married a man she knew was an ISIS member, sent positive tweets about life under ISIS and possessed a firearm.

d. She travelled to Iraq, joined ISIS, adopted the radical and extremist ideology of the armed jihadist struggle of that organisation and possessed a firearm.

e. No verdict was published on the official Dutch judiciary website (https://www.rechtspraak.nl/) regarding this woman’s trial procedure and verdict. Consequently, specific details regarding her alleged terrorist behaviours, which authors could extract from her forensic mental health report, were not included in this paper to prevent traceability.

f. She made arrangements to obtain the necessary funds for, or help with, her journey to an area controlled by ISIS.

g. She made preparations to travel to an area controlled by ISIS, contacted and communicated with ISIS members in Syria and Iraq, booked a flight to Istanbul and packed her suitcase and ID.

h. She travelled to Syria, married a man there, moved to an ISIS-controlled area, joined ISIS, remarried twice through an ISIS marriage bureau after her first husband’s death, lived in ISIS-funded women’s houses during her stay in ISIS territory, engaged in conversations and shared images glorifying life in the conflict zone and managed a chat group where weapons were traded.

i She travelled to Syria, joined ISIS, facilitated her husband who was a member of ISIS, carried out ISIS propaganda on Facebook, attempted to persuade others to travel to Syria and possessed firearms.

j. The same explanation under ‘e’ applies to this woman.

k. She travelled to Syria, joined ISIS, was married to two ISIS fighters, participated in an ISIS chat group and offered hand grenades for sale within it.

l. She gathered information about participating in ISIS and attempted to drive her car to the Turkish border, intending to cross into Syria, but failed.

m. She travelled to in Syria/Iraq, joined ISIS, provided others with intelligence on killing infidels, carrying out attacks, making explosives and bomb belts via social media, and distributed extremist jihadist material on various Telegram accounts.

n. She travelled to Syria, married an ISIS fighter, joined ISIS, carried out propaganda through messages with ISIS-related content and facilitated her husband in his ISIS activities.

### Outcome measures

Based on the aim of our study, we formulated the following four outcome measures: (a) presence and types of diagnosed mental disorders at the time of the terrorist offences; (b) presence and types of diagnosed psychopathological symptoms or pathological personality traits at the time of the terrorist offences; (c) extent of influence exerted on the offences by mental disorders, psychopathological symptoms or pathological personality traits; and (d) description of how the identified mental disorders, psychopathological symptoms or pathological personality traits influenced the commitment of terrorist behaviours among the convicted Dutch women.

### Forensic mental health reports in The Netherlands

As the primary source for retrieval of data regarding outcome measures, the forensic mental health reports of the women in our study sample were utilised (see ‘Data collection and analysis’). In The Netherlands, a forensic mental health report follows a pre-trial assessment by a psychiatrist and/or psychologist – i.e. behavioural expert(s), appointed by the prosecutor, judge or attorney – to evaluate a suspect’s mental state potentially influencing their alleged offences.^25,26^ The pre-trial forensic mental health assessment helps courts determine the suspect’s mental state, impacting decisions on criminal responsibility, reoffending risk and treatment. There are different types of assessments – i.e. monodisciplinary (by a psychiatrist or psychologist) or multidisciplinary (including both, possibly with a report by a social worker about the suspect’s background in a ‘triple report’) – depending, among other factors, on the expected mental disorder(s) in a suspect. In multidisciplinary assessments, consensus is sought but documented disagreements are allowed. In our study, ten women had triple assessments, three had psychological reports with social worker input and one had a psychological report only, all achieving consensus in multidisciplinary cases.

Regarding the content of forensic mental health reports, these follow a standard format and address two key questions, among others^25^: did the suspect have a mental disorder, intellectual disability or psychogeriatric condition at the time of the alleged offence(s)? If so, how can this be described diagnostically? And, did this condition impact the suspect’s behaviour during the alleged offence(s)? If yes, to what extent and how? Behavioural experts assess the influence of psychopathology on offences using a three-point scale: ‘no influence’, ‘partial influence’ or ‘full influence’, leading to a conclusion of ‘full criminal responsibility’, ‘diminished criminal responsibility’ or ‘insanity’, respectively. As noted earlier, the last of these is rare – i.e. it is rare for any mental disorder to entirely determine offending behaviour (see ‘Current empirical understanding of psychopathology in terrorism’).

### Data collection and analysis

Access to the pre-trial forensic mental health reports of the 14 Dutch female terrorist convicts was requested and obtained from the Department of Science and Education of the NIFP (see ‘Ethics approval’). Data for the outcome measures were extracted from two main chapters within the standard structure of each report: the ‘Diagnostic considerations’ chapter, which details diagnosed mental disorders, psychopathological symptoms and pathological personality traits, if any (first and second outcome measures); and the ‘Forensic considerations’ chapter, which details the extent (third outcome measure) and role (fourth outcome measure) of psychopathology in the suspects’ behaviours during the offences, if any. The data presented in this paper are derived from the forensic mental health reports conducted by the examining psychiatrists and psychologists and not by the authors of this study.

More specifically, the diagnoses of mental disorders were derived from Diagnostic and Statistical Manual of Mental Disorders (DSM) classifications, integral to the ‘Diagnostic considerations’ chapter. It is worth noting that, under the DSM, personality disorders are classified as mental disorders. Detected psychopathological symptoms and pathological personality traits were collected by coding the entire text in the aforementioned chapter; for example, the behavioural expert’s statement that ‘the subject exhibits a weak identity’ was coded as ‘identity problems’. Atlas.ti Windows (version 22.0.6.0), a qualitative research tool for coding and analysing text developed by ATLAS.ti Scientific Software Development GmbH, located in Berlin, Germany, was used for this coding process (https://atlasti.com/). The extent of the influence of psychopathology on terrorist behaviours, derived from the ‘Forensic considerations’ chapter, was categorised as either ‘no influence’, ‘partial influence’, ‘full influence’ or ‘no advice’. The last of these categories was used when behavioural experts did not offer advice on the influence – for example, due to a lack of information about the individual’s condition at the time of the offence. Mental disorders, psychopathological symptoms, pathological personality traits and the extent of the influence of psychopathology were quantified at a nominal level (present: yes/no) and statistically analysed using SPSS Statistics (version 25). To describe the role of psychopathology in the terrorist behaviours of a woman, a qualitative approach was employed. This approach involved summarising the role of psychopathology based on the literal descriptions provided by the psychiatrist and/or psychologist in the ‘Forensic considerations’ chapter.

Two independent research assistants (L.v.E. and E.P.), trained by the first author (S.R., a forensic psychiatrist), conducted data collection and statistical analyses. To ensure that the research assistants coded in a manner similar to one another, they initially compared their independently conducted data collection and coding of the outcome measures for two women from the study sample, which showed consistency in their approach. Consequently, each assistant continued with independent data collection and coding of the remaining reports, subsequently comparing her coding with that of the other research assistant. Any discrepancies were to be resolved through consensus between the assistants. In case of disagreement, the first author, being a forensic psychiatrist and behavioural expert, was to be consulted for a definitive decision. However, consulting the first author was never required in our dataset.

### Ethics approval

Because forensic mental health reports and the EDT are not public material, permission to utilise them for our study was requested from, and granted by, the NIFP’s Department of Science and Education and EDT project team, respectively. In its role within the Dutch Ministry of Justice and Security, the NIFP has permission to retrospectively use data from suspects or convicts of offences for scientific research. To ensure the privacy of the subjects, the NIFP employs various measures such as privacy impact assessments (PIAs) and protocols for requesting and providing such data (for the NIFP’s privacy protocol and data request procedure, refer to https://www.nifp.nl/onderwerpen/data-aanvragen-voor-wetenschappelijk-onderzoek/privacy-protocol-en-procedure-data-aanvraag; for details on the EDT’s^19^ data-sharing procedure, refer to https://www.vera-2r.nl/research/eu-database/data-sharing). As stated in the PIA, requesting explicit consent from former suspects or convicts at the time of conducting research is not part of the procedures, because it is often impractical or burdensome. Scientific research involving this population typically includes individuals who are no longer in contact with the NIFP. Obtaining contact details for consent is usually unfeasible and could counterproductively infringe on individuals’ privacy, because former suspects or offenders may have rebuilt their lives or become actively engaged in new pursuits, with their current environment unaware of their past. In addition, seeking consent may result in selective response bias because many (ex-)forensic individuals are likely to ignore consent requests, making robust scientific research nearly impossible. That said, forensic individuals are given the option to ‘opt out’; NIFP brochures with information about pre-trial forensic mental health assessments, distributed by behavioural experts during the initial assessment session, inform participants that they can refuse to allow the use of their anonymised forensic mental health reports in scientific research.

Additionally, we implemented an extra measure to prevent disclosing information about the subjects in our (small) study sample. This was deemed necessary because, when searching for the women’s judicial decisions on the Dutch government’s publicly available judiciary website (see ‘Study sample’), it became clear that the verdicts, in addition to the judicial rulings, included relatively extensive information on the women’s psychopathology and its influence on their terrorist offences (in cases where such conditions were identified by the behavioural experts). To prevent revealing information that could, when combined with the already publicly available data, be traceable to an individual in the study sample, it was ensured that unpublished data from the forensic mental health reports – i.e. data not available on the publicly accessible government website – were not included in our study. It should be noted that this precaution did not impose any limitations on addressing the research questions of the present study. Finally, the entire study was ethically reviewed and approved by the Ethics Committee for Pedagogical Sciences at Leiden University (project no. ECPW-2021/329).

## Results

The sociodemographic characteristics of the study group (*N* = 14) are shown in Table 2.

**Table 2:** Sociodemographic characteristics of Dutch female terrorist offenders (*N* = 14)

Characteristics	Dutch female terrorist offenders
	*N* **(%)**
Age (years)	
Minor (<18)	3 (21)
Adult (≥18)	11 (79)
Relationship status	
Partnered	2 (14)
Married	3 (21)
Divorced	2 (14)
Single	7 (50)
Mother of child(ren)	5 (36)
Religion	
Islam	14 (100)
Education^a^	
No diploma	0 (0)
Lower education	2 (14)
Lower secondary education	7 (50)
Higher secondary education	5 (36)

Data included were correct at the time of the terrorist offences.

a. The highest achieved education level is based on the Dutch system: lower education (in Dutch, Basisschool); lower secondary education (in Dutch, HAVO-VWO onderbouw, VMBO, MBO 1); higher secondary education (in Dutch, MBO 2,3,4, HAVO-VWO bovenbouw).

### Mental disorders

A mental disorder present at the time of the offence(s) was diagnosed in seven female terrorist offenders (50%) (Table 3). The most commonly diagnosed mental disorder was personality disorder – i.e. avoidant and dependent personality traits (*n* = 1), cluster B traits (*n* = 1) and non-specified traits (*n* = 1). Moreover, comorbidity was present in two women, one with a not otherwise specified personality disorder and a somatisation disorder, and one with an autism spectrum disorder and an intellectual disability.

**Table 3:** Mental disorders among Dutch female terrorist offenders and their influence on terrorist behaviours (*N* = 14)

Disorder	Dutch female terrorist offenders			
	Total group	Partial influence	No influence	No advice
	*n* **(%)**			
	14 (100)	6 (100)	7 (100)	1 (100)
Mental disorder	7 (50)	4 (67)	2 (29)	1 (100)
Personality disorder	3 (21)	2 (33)	1 (14)^a^	0 (0)
Avoidant and dependent personality traits	1 (7)	1 (17)	0 (0)	0 (0)
Unspecified personality disorder, cluster B traits	1 (7)	1 (17)	0 (0)	0 (0)
Personality disorder not otherwise specified	1 (7)	0 (0)	1 (14)^a^	0 (0)
Autism spectrum disorder	1 (7)	1 (17)^b^	0 (0)	0 (0)
Bipolar disorder	1 (7)	0 (0)	0 (0)	1 (100)
Depressive disorder	1 (7)	1 (17)^c^	0 (0)	0 (0)
Somatisation disorder	1 (7)	0 (0)	1 (14)^a^	0 (0)
Attention-deficit disorder	1 (7)	0 (0)	1 (14)	0 (0)
No mental disorder	7 (50)	2 (33)	5 (71)	0 (0)

a. These data concern the same woman – i.e. diagnosed with a personality disorder not otherwise specified and a somatisation disorder.

b. In this subject, a mild intellectual disability was also diagnosed.

c. In this subject, the behavioural experts found that the (so-called) threatened personality development, and not the depressive disorder, had influenced her terrorist behaviour.

### Psychopathological symptoms and pathological personality traits

In all 14 female terrorist offenders, psychopathological symptoms were reported by the behavioural experts (Table 4). The most commonly reported psychopathological problems were susceptibility to influence, identity problems, feelings of inferiority and naivety. Borderline and narcissistic personality traits were the most common personality traits (Table 5).

**Table 4:** Psychopathological symptoms among Dutch female terrorist offenders and their influence on terrorist behaviours (*N* = 14)

Symptoms	Dutch female terrorist offenders			
	Total group	Partial influence	No influence	No advice
	*n* **(%)**			
	14 (100)	6 (100)	7 (100)	1 (100)
Psychopathological symptom	14 (100)	6 (100)	7 (100)	1 (100)
Susceptibility to influence	10 (71)	6 (100)	4 (57)	0 (0)
Identity problems	9 (64)	5 (83)	4 (57)	0 (0)
Feelings of inferiority	8 (57)	5 (83)	3 (43)	0 (0)
Naivety	7 (50)	3 (50)	4 (57)	0 (0)
Limitations in social interaction	6 (43)	3 (50)	2 (29)	1 (100)
Limited introspective ability	6 (43)	3 (50)	2 (29)	1 (100)
Impulsivity	6 (43)	3 (50)	2 (29)	1 (100)
Unstable romantic relationships	6 (43)	3 (50)	2 (29)	1 (100)
Sensation seeking	6 (43)	3 (50)	2 (29)	1 (100)
Social isolation	6 (43)	2 (33)	4 (57)	0 (0)
Feelings of sadness	6 (43)	2 (33)	4 (57)	0 (0)
Aggression-regulation problems	5 (36)	4 (67)	0 (0)	1 (100)
Problems with self-direction	5 (36)	2 (33)	3 (43)	0 (0)
Rigidity	5 (36)	4 (67)	1 (14)	0 (0)

**Table 5:** Pathological personality traits in Dutch female terrorist offenders (*N* = 14)

Personality traits	Dutch female terrorist offenders
	*n* **(%)**
Pathological personality traits	
Borderline personality traits	4^a^ (29)
Narcissistic personality traits	3^a^ (21)
Antisocial personality traits	2 (14)
Histrionic personality traits	1 (7)

a. In two of the four women with borderline personality traits, and in one of the three women with narcissistic personality traits, the forensic mental health experts identified these traits but did not diagnose a personality disorder, because the criteria for Diagnostic and Statistical Manual of Mental Disorders classification were not sufficiently met.

### Extent of influence of psychopathology on terrorist behaviours

In almost half of the women (*n* = 6, 43%), mental disorders, psychopathological symptoms or pathological personality traits were judged by the behavioural experts to have partially influenced their terrorist offending behaviours (Tables 3 and 4). As shown in Table 3, in four of these women a mental disorder was diagnosed and associated with terrorism. Although the other two women were not diagnosed with a mental disorder according to DSM criteria, they exhibited borderline personality traits that were not sufficient for a diagnosis of a personality disorder but that were influential in their terrorist behaviours. In contrast, behavioural experts found that in two female subjects diagnosed with mental disorders – i.e. a personality disorder not otherwise specified combined with somatisation disorder in one woman and attention deficit disorder in the other – their criminal behaviour was not influenced by their mental disorders. In these individuals, it was determined that they had retained sufficient agency to make behavioural choices. In the case of one female terrorist offender, no advice on the potential influence of the diagnosed bipolar disorder on her offending behaviour was given due to a lack of collateral information about her psychological condition at the time of her terrorist actions. Lastly, the behavioural experts did not suggest a ‘full influence’ of psychopathology leading to a conclusion of ‘insanity’ in any of the women within the study group (Tables 3 and 4).

### Mechanisms of influence of psychopathology on terrorist behaviours

All six women with identified influence of psychopathology on their terrorist behaviours were susceptible to influence (Table 4). Five of them also exhibited identity problems and feelings of inferiority. The two women without a diagnosed mental disorder but with borderline personality traits contributing (partially) to their offences showed susceptibility to influence, identity problems, feelings of inferiority and impulsivity. Notably, problems with aggression regulation were detected only in the ‘partial influence’ group and in one woman for whom no advice was provided, but not in those in the ‘no influence’ group.

In the following section, summaries of how psychopathology influenced the terrorist activities of the six women, as identified by the behavioural experts, are provided. This section outlines key psychopathological factors affecting terrorist behaviours. Although the summaries do not cover the full forensic analyses, they illustrate how psychopathology is thought to have influenced the women’s terrorist actions.

- One female offender with a personality disorder with cluster B traits, convicted of ‘participation in a terrorist organisation’ and ‘preparation for murder, manslaughter, arson, explosion, occasion, means or intelligence, with terrorist intent’, showed significant naivety and susceptibility. Her journey to Syria was not the result of a religious path she had followed. Her naivety, susceptibility, craving for attention and tendency to seek excitement stemming from her personality structure appeared to have significantly influenced her decision to leave. This aligned with the influence of a man with whom she was in a dysfunctional relationship and who had given her attention, promised her a prosperous future and persuaded her. It seemed she had embarked on an adventure without proper knowledge. (Regarding the actions committed during her stay in Syria, the behavioural experts concluded that they had not obtained sufficient objective insight into her behaviours and life during that period to draw definite conclusions. It was assumed that her diagnosed personality disorder undoubtedly influenced the choices she made in Syria, but the extent and manner of this influence could not be determined with certainty.)
- A female offender of terrorism, diagnosed with borderline personality traits and convicted of ‘participation in a terrorist organisation’ and ‘preparation for murder, manslaughter, arson, explosion, occasion, means or intelligence, with terrorist intent’ grew up in a family situation characterized by role reversal, where she took on responsibilities that belonged to her parents. As a result, she became less in touch with her own emotions, vulnerability and inner world and developed a weak identity. Additionally, she was extroverted and impulsive by temperament, experienced rapid mood swings and had difficulty regulating her anger. Due to a combination of her temperament and the family situation in which she grew up, she exhibited borderline personality traits, such as quick temper, a tendency toward black-and-white thinking, overestimating herself, being overwhelmed by her own emotions and not knowing how to handle them. At the time she travelled to Syria there were various problematic areas in her personality, such as an unstable sense of identity, repressed emotions of fear and insecurity, impulsivity and inner turmoil. From these experiences, she sought strict religious behavioural rules to counterbalance her intense temperament, emotional tensions and need for stimuli, including sexual stimuli.
- One female offender of terrorism, diagnosed with borderline personality traits, convicted of ‘participation in a terrorist organisation’, was born with a physical disability, was adopted by her aunt and, partly due to this early adversity, had a turbulent youth with little room for self-development. In conjunction with her physical limitation, she was emotionally unstable and insecure, which hindered her identity formation. As a result, her personality development was flawed, resulting in a vulnerable and poorly integrated personality with borderline traits. Due to her identity issues, she was susceptible and easily influenced by external factors. Searching for fulfilment and connection, she found it in her quest for the ISIS caliphate. Seeking her own identity, meaning and acceptance, she travelled to ISIS territory.
- One female offender of terrorism, diagnosed with a personality disorder with avoidant and dependent traits and convicted of ‘participation in a terrorist organisation’ and ‘preparation for murder, manslaughter, arson, explosion, occasion, means, or intelligence, with terrorist intent’, had a troubled past with an unstable home situation, resulting in a strong need for control and certainty as well as a desire for clear rules and approval. She developed an immature identity and low self-esteem. Due to her avoidant and dependent personality traits, she repeatedly found herself in harmful interpersonal relationships, further damaging her self-image. She was searching for meaning, direction in her life, guidance and leadership. What appealed to her in Islam were the many strict rules. Despite being an (above)-average intelligent woman who was aware that it was forbidden to travel to Syria, she consciously chose to go, driven by religious and possibly ideological convictions. Due to her dependent and avoidant traits, she was susceptible and receptive to strict fundamentalist ideologies.
- One female offender of terrorism, diagnosed with a light depressive disorder and threatened personality development and convicted of ‘attempted participation in a terrorist organisation’ struggled with significant identity issues, experienced a disrupted personality development and was highly susceptible to influence. In her search for identity, she rigidly attached herself to certain views, people and behaviours, leading to her conversion to Salafism. She harboured aversion towards the West and developed fundamentalist ideas about the interpretation of Islam, resulting in her rejection of democracy. At the time of preparing for her departure towards the ISIS region, she seemed to have lost her way and found herself drawn into extremist ideologies more and more. Her personality and interest in Islam provided a perfect breeding ground for the terrorist ideology of ISIS, leading to further radicalisation.
- In one female offender of terrorism, diagnosed with an autism spectrum disorder and a mild intellectual disability and convicted of ‘participation in a terrorist organisation’, the verdict was not published on the public website of the Dutch government. In order to minimise the risk of traceability, the mechanism through which the diagnosed psychopathology had influenced her terrorist offending behaviour, as described in her forensic mental health report, is not given in this paper. However, to provide an impression of how autism spectrum disorder may shape vulnerability and risk of extremism, some key features will be briefly mentioned here.^14^
- Individuals with autism spectrum disorder may develop intense, obsessional interests, including terrorism-related topics, which can lead to risky behaviours. In addition, their fantasy life, if rewarding and addictive, can drive distress or anger. Social difficulties may push them online, where extremist narratives could be taken literally and reinforce their beliefs. Furthermore, a need for order can make extremist ideologies appealing, and difficulties in understanding implicit agendas may increase their susceptibility to radicalising material.

## Discussion

Empirical research of psychopathology in perpetrators as a risk factor for terrorist behaviour has so far been primarily focused on male offenders. In addition, most existing studies are subject to substantial methodological limitations – e.g. the use of secondary sources rather than rigorous forensic mental health assessments by psychiatrists or psychologists – as a result of which, the questions of whether psychopathology plays a role in terrorist behaviours and, if so, which psychopathological effects underlie this role, have remained largely unanswered. Our study focused on 14 cases of Dutch female offenders of terrorism related to ISIS and investigated, using forensic mental health reports as primary sources, the occurrence and types of psychopathology and – more importantly – the potential link between psychopathology and terrorist behaviours in these women.

In our Dutch sample of women convicted of ISIS-related terrorist activities, our analysis revealed that – according to the behavioural experts – 50% had a mental disorder at the time of their terrorist offending. Besides demonstrating that mental disorders are present among female perpetrators of ISIS-related terrorism, this finding reveals a prevalence rate notably higher than the 17% typically found in predominantly male terrorist populations.^12,13^ However, Merari and Ganor^27^ reported a prevalence of 80% of diagnosed mental disorders in their subsample of Palestinian women with terrorist behaviours. Given these discrepancies in prevalence rates, several challenges need to be addressed when comparing our results with those of existing studies. When comparing our study with those focusing on (predominantly) male terrorist populations, the latter are largely based on secondary sources whereas our study relies on primary data from forensic mental health reports prepared by psychologists and psychiatrists who directly examined female subjects. Open-source studies, while informative,^28^ may underreport the prevalence of mental disorders compared with those involving direct examinations by behavioural experts.^12^ Additionally, even diagnostic assessments by healthcare professionals can be influenced by gender biases, particularly the tendency to perceive violent women as mentally ill compared with their male counterparts.^29^ Comparing our findings with studies featuring female-specific data based on primary sources also presents difficulties due to the scarcity of such studies^15^, as well as complexities arising from comparing female perpetrators across different types of terrorism and cultural contexts, which need to be considered when studying the pathways to terrorist behaviours.^30,31^ For instance, our Dutch sample comprises Western women recruited online for group terrorism related to ISIS, while Merari and Ganor’s^27^ sample consists of Palestinian paramilitary women independently carrying out terrorist attacks. Despite these challenges, and with appropriate caution, our study suggests a potentially higher occurrence of mental disorders among women involved in ISIS-related terrorism compared with the general male terrorist population.

In addition to mental disorders, our study explored the occurrence of psychopathological symptoms and pathological personality traits in the female sample, finding some form of psychopathological issue present in all the women. Although behavioural assessments might identify some psychopathological issues even in psychologically healthy individuals, this finding remains particularly relevant because pathological personality traits in two women in our study, despite not meeting the full criteria for a personality disorder, were found to have played a significant role in their terrorist offences. This finding underscores the importance of considering subthreshold psychopathological phenomena in terrorist perpetrators – pathological personality traits in particular – because they might be relevant in pathways towards the commission of terrorism.^15,20,21^ Furthermore, it should be acknowledged that the psychopathological problems measured in the present study are also commonly studied as factors unrelated to psychopathology in non-diagnostic contexts. While the pathologisation of such factors should be avoided when they are not related to underlying psychopathology, it should be noted that the symptoms coded as psychopathological in the current study were derived from the DSM and, according to the authors, are diagnostically related to psychopathology. Future research could further explore these factors from a diagnostic perspective, given the limited work in this area.^14^

Next to the presence of psychopathology, our study investigated its types as assessed by the behavioural experts in our study sample. The mental disorder most commonly diagnosed in the Dutch female terrorist offenders was personality disorder. Although comparisons based solely on gender are not justified, personality disorders were also notably reported in Merari and Ganor’s^27^ sample of Palestinian paramilitary women involved in terrorism. In terms of the type of personality pathology, our study revealed that borderline personality traits were the most prevalent. This finding aligns with existing literature, which indicates that borderline personality traits can lead to terrorist behaviours through an unstable identity, emotional fluctuations and a fear of abandonment, with extremist groups consequently offering the clear identity, emotional outlet and belonging sought by individuals with these traits.^14^ In addition to personality disorder, we found a wide range of mental disorders in our study sample – e.g. autism spectrum disorder, attention-deficit disorder, depressive disorder, bipolar disorder and (mild) intellectual disability. These mental disorders correspond with those reported in predominantly male terrorist populations.^13,32^ The psychopathological symptoms most commonly reported in our study group were susceptibility to influence, identity problems, feelings of inferiority and naivety. Remarkably, no instances of suicidality were observed among the women we studied, contrasting with the findings of Merari and Ganor^27^ within their sample of Palestinian women who had carried out attacks against Israeli targets on their own initiative. According to Merari and Ganor, the attacks carried out by the women in their study can be seen as a means for these suicidal individuals to achieve martyrdom, which in Islam promises a glorious place in paradise, unlike ‘ordinary’ suicide, which is believed to lead to hell.^27^ This understanding of suicidality in terrorist perpetrators aligns with theoretical perspectives suggesting that it stems from individuals perceiving they have nothing to lose or seeking a heroic demise imbued with meaning.^14^ The absence of suicidality in the women in our study may in turn be attributed to our focus on female terrorist offenders engaged in roles assigned by ISIS, such as wives, mothers, educators, recruiters and fundraisers. Although it needs to be empirically validated, the hypothesis that such roles attract women who are susceptible to influence, grappling with identity issues and seeking a sense of purpose through active roles promoted by ISIS, rather than those who are suicidal, deserves consideration.

Investigating the link between psychopathology and terrorist behaviour as the major focus of our study, it was revealed that, in about half of the Dutch female ISIS affiliates, the behavioural experts found that psychopathology had an influence on their terrorist behaviours. This is an empirical novel finding. Susceptibility to influence and identity problems were the most common psychopathological mechanisms of influence, along with other features such as feelings of inferiority, naivety and impulsivity. Interestingly, as mentioned earlier, in two women who did not meet DSM criteria for a personality disorder and with significant pathological borderline personality traits, these traits were assumed to have played a role in their terrorist behaviours. Conversely, in two female subjects diagnosed with a mental disorder, the behavioural experts found no discernible influence of the diagnosed mental disorders on their terrorist offences. Thus, while our study confirms previous research^12,13,32^ indicating that psychopathology does not necessarily play a significant role in terrorist behaviours of individuals in whom it is diagnosed, it also shows that psychopathology can be relevant in some individuals – not as the sole factor but as part of a cascade of personal, social, and contextual influences – and therefore should be examined on a case-by-case basis.

The present study enhances our empirically based understanding of the presence and relevance of psychopathology among the understudied population of female perpetrators of terrorism. Specifically, within a Dutch sample of women involved in ISIS-related terrorism, the study shows that psychopathology is both present and relevant in some women while for others it is either not present or not relevant despite being present. As aptly stated by Gill and colleagues^13^ ‘rarely are mental health problems the sole issue [in the pathways leading to terrorist behaviour]’. Concerning psychopathology, psychiatrists – regardless of their patient population(s) or area(s) of expertise – should be knowledgeable about common psychopathological issues in female terrorist perpetrators because they may encounter women (and girls) at risk of radicalisation or potential terrorist behaviour in their practice. Early identification and prevention of such risks should be integral to every psychiatrist’s core competencies. This paper may serve as a resource for psychiatrists, psychologists and other healthcare professionals working with this group by providing insights into the mental disorders, psychopathological issues and pathological personality traits associated with terrorist behaviours in women.

While our study offers unique and empirically based insights into the complex relationship between psychopathology and terrorist behaviours among female perpetrators, further research is essential to build on these findings. Our current sample, although informative, is limited in both size and scope, focusing specifically on 14 Dutch female convicts involved in group terrorist behaviours related to ISIS. To address these limitations, future studies should involve larger female cohorts and compare them with female groups engaged in other types of terrorist violence and non-terrorist violence, and male terrorist groups. This expanded scope will provide a more comprehensive understanding of the psychopathological characteristics relevant to female terrorist perpetrators across different contexts. Additionally, incorporating a broader range of personal, psychological and contextual data will facilitate the early identification of women at risk of engaging in terrorist activities and allow for timely, targeted interventions. This approach will also help dispel gender stereotypes and offer a more nuanced understanding of the motives and behavioural (dys)functioning of women involved in terrorist violence, which are often oversimplified. Ultimately, such knowledge is expected to enhance the development of tailored, highly individualised, female-centred counterterrorism interventions aimed at reducing the risk of both new and recurring terrorist attacks and contributing to global security.

### Limitations

While the use of primary-sourced materials is considered a strong feature of the present study and allows for an empirically based exploration of the presence and relevance of psychopathology in female perpetrators of ISIS-related terrorism, the findings should be interpreted with caution due to the following limitations. First, the very reliance on forensic mental health reports might have introduced several biases. Although behavioural experts are trained to distinguish between suspects’ subjective and objective data from their own forensic mental assessments, it is conceivable that suspects may have emphasised mental health problems to mitigate criminal responsibility. This, in turn, could potentially have led to a distorted portrayal of their mental state, resulting in an overestimation of the prevalence or relevance of psychopathology in the present study. More specifically, the female subjects in our study might have claimed specific psychopathological issues, such as those indicating susceptibility, aiming ultimately to obtain leniency and a lighter sentence. The objectivity of the forensic mental health assessments might also have been compromised by potential biases of the behavioural experts regarding gender perceptions, particularly the perception of female perpetrators as vulnerable.^29,33^ Moreover, variations in approaches, tests and diagnostic perspectives among behavioural experts could have affected their forensic interpretations. Additionally, mental health assessments are particularly complex due to the retrospective nature of evaluating defendants’ mental states at the time of the offence. This complexity is further heightened by the potential development of psychopathological issues post offence, especially in traumatic environments such as those associated with regions controlled by ISIS. Also, the present study did not include a control group, making it difficult to explore a potential causal relationship between psychopathology and terrorist behaviours; without a control group, it is challenging to determine whether the observed outcomes are due to the risk factor being investigated – i.e. psychopathology – or other concurrent variables – e.g. perceived discrimination in offenders. Lastly, the small size of the study sample and its focus on a specific type of terrorist offences limit the generalisability of the findings to the broader female terrorist population.

## Data Availability

The data that support the findings of this study are available on reasonable request from the corresponding author, S.R.
