# Psychopathology in Dutch women with terrorist behaviours: empirical case series study – CORRIGENDUM

**DOI:** 10.1192/bjo.2025.10063

**Published:** 2025-05-26

**Authors:** Sadaf Rakhshandehroo, Nils Duits, Lieke van Emmerik, Elise Pullen, Robbert-Jan Verkes, Maaike Kempes

In the original publication of this article, the co-author Lieke van Emmerik’s name was misspelled as ‘Lieke van Emmeriek’.

The article has now been updated and this corrigendum published.
